# Lifespan Psychomotor Behaviour Profiles of Multigenerational Prenatal Stress and Artificial Food Dye Effects in Rats

**DOI:** 10.1371/journal.pone.0092132

**Published:** 2014-06-17

**Authors:** Zachary T. Erickson, Erin A. Falkenberg, Gerlinde A. S. Metz

**Affiliations:** Canadian Centre for Behavioural Neuroscience, University of Lethbridge, Lethbridge, Alberta, Canada; University of Insubria, Italy

## Abstract

The consumption of artificial food dye (AFD) during childhood and adolescence has been linked to behavioural changes, such as hyperactivity. It is possible that the vulnerability to AFDs is modified by prenatal stress. Common consequences of prenatal stress include hyperactivity, thus potentially leading to synergistic actions with AFDs. Here, we investigated the compounding effect of multigenerational prenatal stress (MPS) and AFD consumption on the development of hyperactivity and anxiety-related behaviours across the lifespan in male rats. MPS treatment involved a family history of four consecutive generations of prenatal stress (F4 generation). AFD treatment included a 4%-concentration of FD&C Red 40, FD&C Yellow 5, FD&C Yellow 6, and FD&C Blue 1 in the drinking water from postnatal days 22 to 50 to resemble juvenile and adolescent dietary exposure. Using several exploration tasks, animals were tested in motor activity and anxiety-like behaviours from adolescence to 13 months of age. MPS resulted in hyperactivity both early (50 days) and later in life (13 months), with normalized activity patterns at reproductive age. AFD consumption resulted in hyperactivity during consumption, which subsided following termination of treatment. Notably, both MPS and AFD promoted risk-taking behaviour in young adults (3 months). There were few synergistic effects between MPS and AFD in this study. The findings suggest that AFDs exert the most noticeable effects at the time of exposure. MPS, however, results in a characteristic lifespan profile of behavioural changes, indicating that development and aging represent particularly vulnerable periods in life during which a family history of prenatal stress may precipitate.

## Introduction

During critical periods of neurodevelopment, both before and after birth, environmental and endocrine factors can have long-lasting effects on brain plasticity and behaviour. One of the most critical influences on neurodevelopment is prenatal stress. Prenatal stress will expose the developing fetal brain to elevated levels of glucocorticoid hormones, which may lead to increased risk of psychological disruptions later in life, including hyperactivity and anxiety-related behaviours [1]–[4]. These stress-induced behavioural changes may be influenced by an organism's family history as well, as many behavioural effects of stress may be transmitted from one generation to the next [5]–[7]. In a continuously stressful environment, however, prenatally stressed females may themselves be exposed to stress during pregnancy, thus generating several generations of prenatally stressed offspring. While the significant sequelae of prenatal stress in a single generation have been investigated in detail, the influence of multigenerational prenatal stress (MPS) on behaviour and brain development have not been shown yet. In turn, such family history of prenatal stress may also raise the vulnerability to environmental risk factors [8].

An environmental risk factor that was suggested to influence behavioural development is the consumption of artificial food dyes (AFDs). Although the contribution to behavioural disturbances is still controversial [9], evidence suggests that AFD consumption increases the risk of behavioural change in children [10]–[13]. Earlier reports indicated that children with ADHD may show above-average sensitivity to AFDs [14], while other findings suggested that AFDs may affect healthy populations as well [15]. In particular, previous studies showed that AFD consumption results in increased motor activity [15]–[16]. For example, a randomized study showed that AFD exposure in 3-year old and 8/9-year old healthy children results in hyperactivity, with the greatest effect in the 8/9-year age group [17]. Very few studies, however, used animal models to pursue further mechanistic studies of the behavioural effects of AFD exposure [18].

The purpose of this study was to examine the effects of MPS and chronic AFD consumption during adolescence on locomotor activity and anxiety-like behaviours in aging rats. We hypothesized that both MPS and AFDs represent experiences that may modulate brain development in adolescence and alter lifespan behavioural profiles in an age-dependent manner. The study used a standard exploratory task (open field) and a newly developed test of affective state and exploration. The purpose of this study was (1) to investigate the behavioural consequences of MPS, (2) to investigate the behavioural effects of chronic consumption of commonly used certified AFDs during neurodevelopment, and (3) to determine potential synergistic effects of MPS and AFDs from adolescence to late adulthood.

## Methodology

### Animals

Thirty-two male Long-Evans hooded rats, weighing an average of 46 g at the beginning of the study, were used. The animals were housed in pairs in standard shoebox polycarbonate cages on corn cob bedding (Bed O Cobs 1/8″, Anderson, OH, USA). The housing room was maintained at 20°C and relative humidity at 30% on a 12-hour dark/light cycle with light starting at 7:30 AM. The experimental procedures were approved by the University of Lethbridge Animal Welfare Committee according to guidelines set forth by the Canadian Council on Animal Care.

### Experimental Design

**Figure 1:**
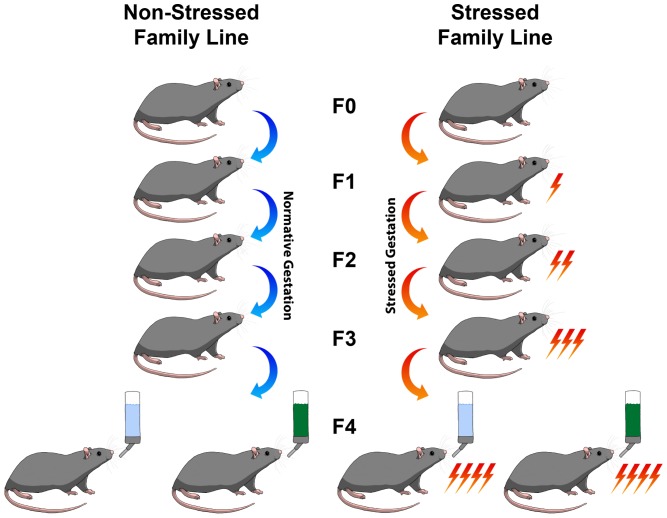
Experimental design. The present study used male rats in a two-by-two factorial design. The independent variables were multigenerational prenatal stress and artificial food dye (AFD) consumption. The multigenerational prenatally stressed rats were the fourth generation (F4) of a familial line in which dams in each generation were given stress during pregnancy. Both the stressed and non-stressed animals were given either normal tap water or a 4%-AFD solution for regular consumption.

The present study was conducted with a two-by-two factorial design, with multigenerational prenatal stress (“Stress”) and adolescent consumption of artificial food dye (“Dye”) as the two treatments (see Figure 1). The male F4 generation born to four generations of stressed dams was used in this study. The experimental design resulted in four groups of rats (n = 8 per group): (1) non-treated controls; (2) Stress without Dye consumption; (3) Dye consumption without Stress; (4) Stress combined with Dye consumption. Each group included offspring from four different litters (n = 2/litter/group). Dye solution was provided from postnatal day (P) 22 (infancy) to P 50 (adolescence). At P 50, all animals given the dye treatment were switched to standard tap water. The animals were allowed to drink *ad libitum*, and fluid consumption and body mass were monitored daily.

Animals were tested in locomotor activity and emotional behaviours at P 50, and at the age of 3 months, 7 months, and 13 months. Behavioural analyses were performed by an experimenter blind to the experimental conditions.

### Multigenerational Prenatal Stress

Four consecutive generations of rat dams were stressed daily during pregnancy from gestational day 12 to 18 [7]. The stress treatment involved two different stressors, which previously have been validated as effective stress procedures [19],[20]: (1) restraining the dam in a Plexiglas tube for 20 minutes and (2) placing the dam in a barrel of room temperature water (∼25°C) for 5 minutes. The dams received each of the two treatments daily at 08:00 or 16:00, and varied in a semi-random fashion to avoid habituation. As in the F0–F2 generations before, nulliparous females from the F3 generation were bred with naïve, unstressed young adult Long-Evans males (obtained from Charles River Laboratories International Inc., Wilmington, MA, USA) to generate the F4 generation. Breeding occurred between the age of P 110 and P 180. Dams only delivered a single litter before being euthanized. Litters were divided equally into AFD and non-AFD treated groups. The pups were weaned at P 21 and housed in pairs with siblings by sex. Maternal corticosterone levels were monitored before pregnancy, gestational day 11 (before stress), gestational day 18 (the final day of stress), and one day following delivery. Maternal blood sampling occurred between 8:00 AM and 10:00 AM, and no more than 0.6 ml was taken from a rat on each sampling day.

### Artificial Food Dye Treatment

Animals received an AFD solution in place of regular drinking water from P 22 to P 50. The AFD solution consisted of 1 gram per litre of four common AFDs: FD&C Red 40, also known as Allura Red AC; FD&C Yellow 5, also known as Tartrazine; FD&C Yellow 6, also known as Sunset Yellow FCF; and FD&C Blue 1, also known as Brilliant Blue FCF (all from Sigma-Aldrich, St. Louis, MO). Fluid consumption and body mass were monitored and recorded. Acceptable Daily Intake (ADI) in mg/day for each dye was calculated using body mass and suggested levels from the Food & Drug Administration in mg/kg/day [21], and compared to the actual amount of dye consumed daily.

### Behavioural Assessments: Open Field Task

The animals were tested using an open field task [22],[23], at P 50, 3 months, 7 months, and 13 months of age. The open field task allows the quantification of motor activity, anxiety-like behaviours, and exploration in an open arena. In this study, the open field task was conducted using the VersaMax Legacy Open Field activity box (Omnitech Electronics, Inc., Dartmouth, NS, Canada), which measured an animal's activity for 10 minutes using an array of infrared sensors connected to a computer. This test was conducted at P 50, 3 months, 7 months, and 13 months of age.

Behavioural measures included total distance travelled during the testing period (Distance Travelled), the total time spent moving during the test interval (Movement Time), and the amount of time spent within the margins of the open field (Margin Time).

### Behavioural Assessments: Affective Exploration Task

The animals were tested at 1.5 months, 3 months, and 13 months in a new task developed for this experiment, the affective exploration task. This task combines the features of a light-dark test [24] with an open field arena. A rat was placed inside a refuge (a 10 cm×10 cm×20 cm plastic tube attached to a small platform) on the top of a large table (75 cm×150 cm) for 5 minutes. The test was video recorded for later scoring and analysis. After each 5 minute session, the testing environment was cleaned to remove any olfactory cues.

The video footage was scored for three main measures of affective state and exploration: the time before initial emergence from the refuge (Emergence Latency), the total time the rat spent within the refuge (Refuge Time), and number of exits from the refuge (Refuge Exits) were recorded. Finally, each animal was categorized on the basis of whether or not it left the refuge during the testing period (Binary Exploration).

### Statistical Analysis

Collected data were analysed using the SPSS version 20 software package. Primary analysis consisted of analysis of variance (ANOVA) and independent sample t-tests to investigate effects at a post-hoc level. Correlation analyses were also conducted between pairs of variables, controlling for Stress Treatment, Dye Treatment, and Age. In partial correlations, including the affective exploration task, the Binary Exploration Score was also controlled for. A p-value less than 0.05 was considered as significant. All data in figures are shown as mean values and standard error of the mean (SEM).

## Results

### General Observations

An independent sample t-test revealed that there was no significant difference (t(5) = 1.07) in litter size between stressed (M = 14.25, SD = 1.7) and unstressed dams (M = 16.00, SD = 2.6). A one-tailed independent t-test showed that maternal corticosterone levels at gestational day 18 were significantly higher (t(3.49) = −2.39, p<0.05) in stressed dams (M = 1630.58 ng/ml, SD = 579.6) when compared to unstressed control dams (M = 910.03 ng/ml, SD = 146.0). Maternal corticosterone levels did not differ between groups on non-stress days, such as baseline, gestational day 11, or one day following delivery.

The administration of AFDs in the drinking water did not affect daily water consumption or weight gain. Averaged over the last four days of the dye consumption period, rats that consumed dye (M = 48.1 ml, SD = 2.7) did not differ from rats that consumed water (M = 49.7 ml, SD = 5.3; F(1,28) = 1.044, p = 0.32). Similarly, rats that consumed dye (M = 281.7 g, SD = 17.2) did not significantly differ in body mass at P 50 when compared to rats that consumed water (M = 285.5 g, SD = 25.9; F(1,28) = 0.424, p = 0.52). In both cases, stress did not have a significant main effect, and there was no interaction effect with dye. Each dye was ingested on average at a rate of 1.71 mg/day (SD = 1.71). The calculated ADI for each dye were as follows: Allura Red had a mean ADI of 2.0 mg/day (SD = 0.12), Tartrazine had a mean ADI of 1.41 mg/day (SD = 0.08), Sunset Yellow had a mean ADI of 1.06 mg/day (SD = 0.06), and Brilliant Blue had a mean ADI of 3.38 mg/day (SD = 0.21).

### Multigenerational Prenatal Stress and Dye Consumption Alter Open Field Activity Profiles

Overall, the ANOVA revealed a significant main effect of Stress (F(1,125) = 7.11, p<0.01) and Age (F(3,125) = 13.61, p<0.001) for Distance Travelled, as well as a significant main effect for Age in Movement Time (F(3,125) = 19.17,p<0.001). There was no significant interaction between Stress, Dye, and Age. At 1.5 months old, a significant main effect of Stress was present in Distance Travelled (F(1,30) = 8.316, p<0.01) and Movement Time (F(1,30) = 5.61, p<0.05), and a significant main effect of Dye was present in Movement Time (F(1,30) = 4.45, p<0.05). At both 3 and 7 months old, there were no significant main effects. At 13 months old, trends in the main effect of Stress were present in Distance Travelled (F(1,29) = 3.65, p = 0.067) and Movement Time (F(1,29) = 2.83, p = 0.10), but not for the main effect of Dye. Both Distance Travelled and Movement Time showed a characteristic age profile, with Distance Travelled and Movement Time being highest at 3 months postnatal, and lower both before and after that age. It should be noted that no significant effect was found for dye consumption after the animals were returned to standard tap water. Additionally, there were no significant effects in the analysis of Margin Time in the open field.

**Figure 2:**
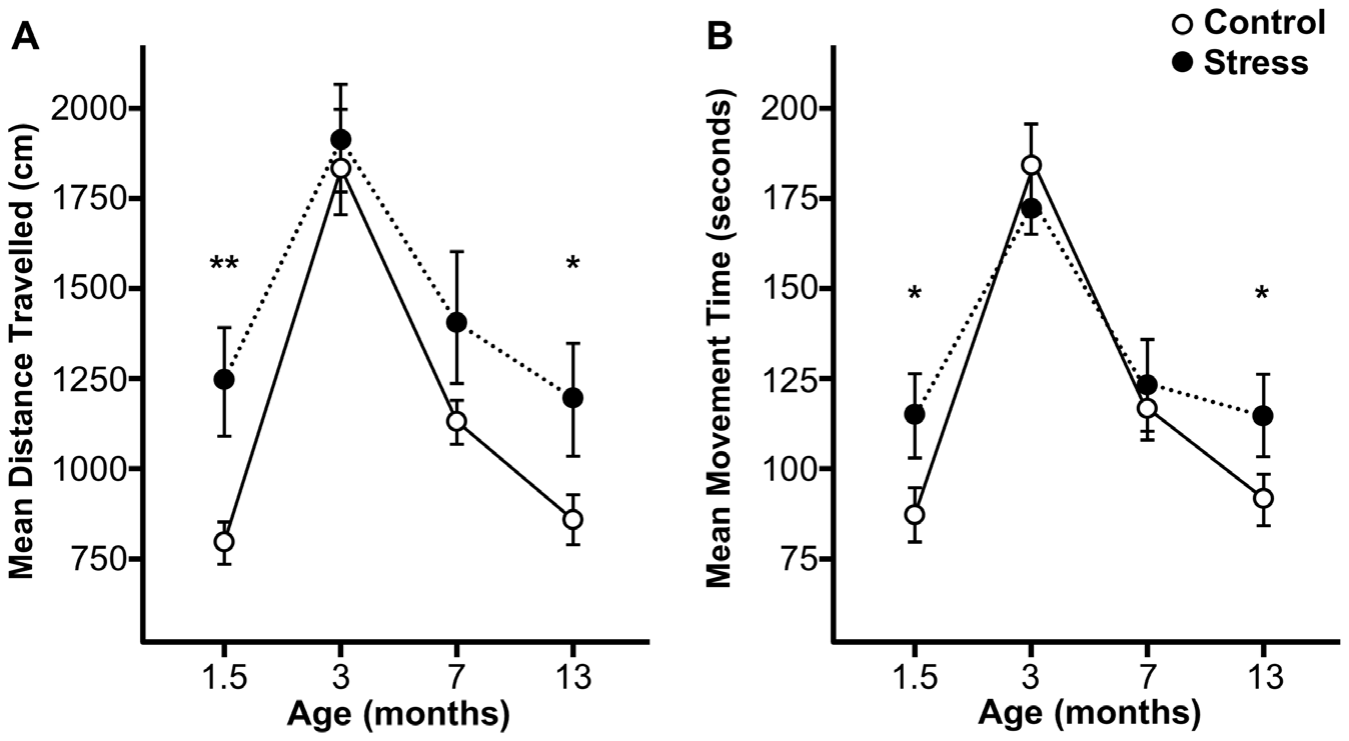
A lifespan profile of the effect of multigenerational prenatal stress on motor activity in the open field task. (A) Multigenerational prenatal stress (n = 16) resulted in a significant increase in the mean distance travelled at 1.5 months and 13 months of age, but not at 3 or 7 months of age, when compared to non-stress animals (n = 16). (B) Multigenerational prenatal stress (n = 16) resulted in a significant increase in mean movement time at 1.5 months and 13 months of age, but not at 3 or 7 months of age, when compared to non-stress animals (n = 16). Thus, motor activity of MPS rats resembles that of non-stressed rats during peak sexual reproductive age, but differs early in life and in late adulthood. Asterisks indicate significances: * p<0.05; ** p<0.01, compared to non-stress animals.

When comparing treatment groups, stress rats, independently of dye treatment, showed significantly higher Distance Travelled at 1.5 months old (t(30) = −2.86, p<0.01) and 13 months old (t(29) = −2.00, p<0.05; one-tailed; see Figure 2A). Stress rats also showed significantly more Movement Time than unstressed controls at 1.5 months old (t(29) = −2.26, p<0.05), and 13 months old (t(29) = −1.75, p>0.05; one-tailed; see Figure 2B).

**Figure 3:**
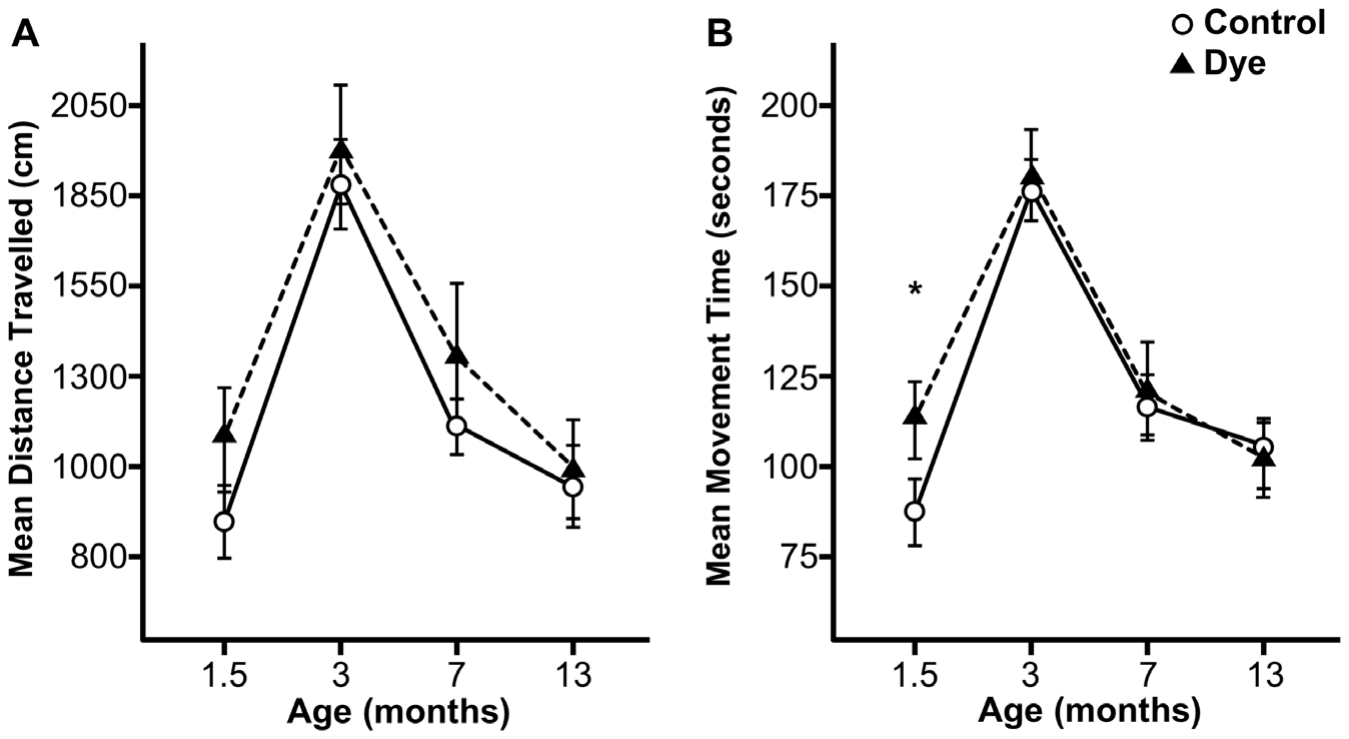
A lifespan profile of the effect of artificial food dye consumption on motor activity in the open field task. (A) AFD consumption (n = 16) from postnatal days 22 through 50 did not increase the mean distance travelled when compared to untreated animals (n = 16). (B) AFD consumption (n = 16) resulted in an increase in the mean movement time while the animals were placed on the AFD-containing diet when compared to untreated animals (n = 16). However, these effects were not found after the AFD was removed from the diet. Asterisks indicate significances: * p<0.05, compared to untreated animals.

There was no difference in dye treatment groups when comparing Distance Travelled (see Figure 3A). However, independently of stress treatment, dye rats showed significantly higher Movement Time at 1.5 months old (following dye consumption) than rats that did not consume dye (t(30) = −1.98, p<0.05; one-tailed; see Figure 3B).

### Dye Consumption Alters Affective Behaviour and Exploratory Behaviours

**Figure 4:**
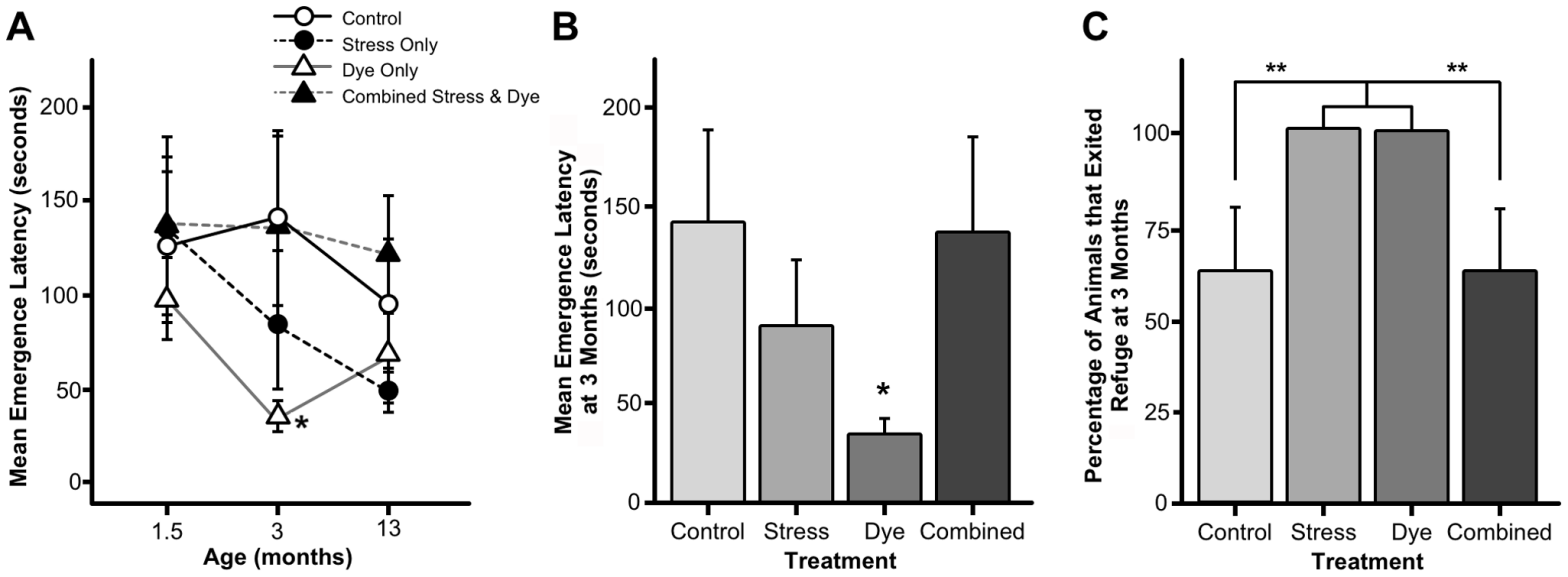
Behavioural effects of multigenerational prenatal stress and artificial food dye consumption in the affective exploration task. (A) At both 1.5 and 13 months of age, there were no effects of treatment with regard to the latency to emerge from the refuge. However, at 3 months of age, AFD-only animals (n = 8) were less reluctant to exit the refuge than any other group (n = 8 per group). (B) At 3 months of age, the AFD-only group (n = 8) was the fastest to exit the safe refuge. (C) Percentage of animals in each group that left the safe refuge at the age of 3 months. Both the prenatally stressed and AFD-treated groups left the refuge in 100% (n = 8) of the test sessions, while both control and combined prenatal stress and AFD groups left the safe refuge only in 62% of the time (n = 5 out of n = 8). Asterisks indicate significances: * p<0.05; ** p<0.01, compared to controls.

When considering Emergence Latency, the ANOVA across the three testing periods showed a significant interaction effect for Stress*Dye (F(1,92) = 5.25, p<0.05; see Figure 4). However, significant main effects, including a main effect of Age, were not found for Emergence Latency when analysing all three testing periods cumulatively. When conducting ANOVA at individual time points, only the 3-month period showed a trend (p = 0.054) for the same Stress*Dye interaction. It is interesting that a significant effect of Dye was found only at the 3-month time point, i.e., 40 days after the cessation of Dye consumption. At 3 months of age, Dye-only animals emerged much faster from the refuge than untreated controls (t(14) = 2.24, p<0.05; Figures 4A and 4B). The analysis of both Refuge Time and Number of Refuge Exits showed no significant effects.

An analysis of the Binary Exploration Score showed a significant interaction effect for Stress*Dye across all ages when controlling for age (F(1,92) = 9.05, p<0.01). When comparing at 3 months of age, a similar interaction effect was found (F(1,31) = 8.40, p<0.01; Figure 4C). More specifically, at 3 months of age, both Stress-only and Dye-only groups left the refuge at a rate of 100%, whereas the control and combined Stress and Dye groups left the safe refuge at a rate of 62.5%.

### Correlations

When controlling for Stress, Dye, Age, and Binary Exploration Score, the negative correlation was significant between Centre Time in the open field and Time Spent in the refuge in the affective exploration task (R^2^ = −0.260, p<0.05). Thus, animals that spent more time in the open field centre also showed the shortest latency to exit the safe refuge in the affective exploration task.

Additionally, the Distance Travelled and Movement Time in the open field were significantly correlated (R^2^ = 0.942, p<0.01), as well as the Refuge Time and Emergence Latency in the affective exploration task (R^2^ = 0.670, p<0.01). Thus, animals that travelled farther in the open field spent more time moving, and animals that spent more time in the refuge also showed the longest emergence latency. All other pairings were not statistically significant, including the pairing of Total Distance and Refuge Time (R^2^ = −0.084, p = 0.51).

## Discussion

The purpose of this study was to investigate the consequences of multigenerational exposure to prenatal stress and AFD consumption during development on the vulnerability to hyperactivity and anxiety-related and risk-taking behaviours in male rats. We found that MPS promoted motor hyperactivity during particularly vulnerable periods in life, during adolescence (P 50) and aging (13 months). Furthermore, AFD consumption from postnatal day 22 to 50 resulted in hyperactivity and reduced anxiety-like behaviour and greater tendency for risk taking in adolescence. The combination of MPS and AFD, however, did not exaggerate anxiety-like behaviours. Interestingly, the MPS animals showed no significant manifestation of anxiety-like behaviour.

In this experiment, pregnant rat dams were exposed to a semi-random combination of restraint and swim stress, which may represent an ecologically valid rat model of moderate stress [7],[19] with effects on motor behaviour [21]–[23]. We used the open field task, a standard test to effectively assess motor activity and anxiety-related behaviour [24]–[25]. Moreover, we developed a new task, the affective exploration task, which is based on a combination of light/dark and open field tasks [26]. Time spent in the refuge in the new affective exploration task was negatively correlated with time spent in the centre of the open field, which serves as a demonstration of its validity to assess anxiety-related and risk-taking behaviours.

The present study explored the effects of multigenerationally recurrent prenatal stress. We hypothesized that the recurrent prenatal stress would enhance the predisposition to hyperactive behaviours and the vulnerability to potential psychomotor effects of environmental compounds, such as AFDs. The exposure of the developing brain to elevated glucocorticoid levels during prenatal stress programs hypothalamic-pituitary-adrenal (HPA) axis activity, which may in turn alter locomotor activity profiles and increase anxiety-related behaviours [3]–[4], [27]. Prenatal exposure to elevated glucocorticoid levels has been shown to alter mesolimbic dopamine (MesoDA) system activity and increase motor activity and ADHD-like behaviours in animal models [28]–[31]. In our study, the MPS rats showed an increase in hyperactivity both in early and late adulthood, suggesting a transgenerationally cumulative programming of the MesoDA system by prenatal stress that becomes exposed during the most vulnerable periods in life.

A common consequence of exposure to prenatal stress in a single generation, in the male F1 generation, is the elevation of anxiety-like behaviours [32]–[34]. The compounding influences of multigenerational exposure to prenatal stress across four generations of individuals have not been previously studied. Although many endocrine and behavioural responses may adapt or become resilient to recurrent mild maternal/prenatal stress across generations [35], we were able to show changes in psychomotor profiles across the lifespan. Interestingly, MPS did not increase anxiety-related behaviour in the open field or the affective exploration tasks, but rather promoted risk-taking behaviours. It is possible that the experience of a continuously stressful environment across generations may promote some form of stress resilience or coping. This is suggested by previous findings that indicated stress-mediated adaptation to a stressful environment [35]–[36]. Moreover, MPS rats may display enhanced anxiety-like responses in other tasks or environments not tested here. Moreover, the behavioural profiles of MPS rats do not exactly resemble those of non-stressed rats either, as seen in previous findings [7]. It is possible that fear-related responses interact with risk-taking behaviours thus masking a conclusive profile of anxiety-like behaviours under the present testing conditions. Regardless, additional research is needed to determine the extent to which MPS and single-generation prenatal stress differ.

Our findings are consistent with previous findings that prenatal stress results in programming effects both early [4],[37]–[38] and later in life [39]. It has been suggested that the deleterious effects of prenatal stress seen in juvenile male rats may serve to eliminate weaker potential mates, resulting in a net benefit toward the survival of the species in a stressful environment [37]. By contrast, early behavioural changes may assist survival until reproduction is secured [4]. Additionally, some of the cognitive effects of prenatal stress may not manifest until later in adulthood [39]. Further studies are necessary to reveal the mechanisms that explain why a family history of prenatal stress resembles non-stressed behaviours during peak sexual reproductive age, but differs both early in life and in late adulthood.

The present AFD administration procedure was based on a previous protocol by Tanaka [40]. In our study, we used FD&C Red 40, FD&C Yellow 5, FD&C Yellow 6, and FD&C Blue 1, which are among the most commonly used AFDs that are approved by the Food and Drug Administration in the United States. Although the AFD-containing drinking water was readily accepted by the animals, the freely available solution in group housing bears limitations by preventing the measurement of individual dose-response relationships. Additionally, it is still unclear if individual AFDs result in interactions when consumed alongside other AFDs [11]. However, it is common for more than one AFD to be used in food products and at concentrations similar to the present ones. It should also be noted, however, that Tartrazine and Sunset Yellow consumption exceeded the ADI in the present study. Given the suggested exponential increase in the average intake of AFDs over the past decades [9], we believe that the present approach may be representative of actual AFD exposure encountered by humans.

Our findings suggest that AFD consumption at least contributes to motor hyperactive behaviours independently of underlying stress-induced programming. These results are similar to other experiments that linked AFD consumption to increased motor activity [15]–[16]. However, an interesting finding of the present study was observed following dye treatment cessation at three months of age in the affective exploration task. While all of the MPS-only and AFD-only rats left the refuge, only two-thirds of the animals treated with combined MPS and AFD left the refuge, consistent with the control group. Based on the parameters collected in the present study an unambiguous explanation of this observation is not possible. One explanation may be that MPS and AFD have synergistic, potentially stimulating effects on cognitive performance, as previously shown for caffeine [41] or L-amphetamine [42] in rat models of ADHD. Additionally, the binary exploration score and activity in the open field were not significantly associated with each other, suggesting that the binary exploration score may not reflect motor hyperactivity but aspects of cognitive functions or fear-related behaviour not assessed in the present study. The leaving of the refuge, however, may suggest a reduced fear of the open, brightly lit surface area in AFD-treated rats.

Although the present study indicates behavioural changes as a function of cumulative AFD consumption as expected to occur in human populations, the use of a cocktail of compounds does not allow dissociation of the effects of individual dyes and their dose-response relationships. Some dyes may be more potent in their actions on behaviour [18],[43] and genotoxicity [11],[39]. A well-studied compound is Allura Red, which produces physical and behavioural toxicity in rats [43]. Notably, these effects can be partially passed on to the offspring and increase post-weaning open field vertical activity [43]. The mechanisms of specific AFDs on behaviour may include modulation of stress response, inhibition of serotonergic activity, and histamine release [9].

The findings of the present study emphasize the critical role of the early environment on behavioural development, adulthood and aging. An important observation is that motor activity profiles of MPS rats resemble those of non-stressed rats during the peak sexual reproductive age, but differ during the most vulnerable periods in life, during development and aging. Although long-term consequences of prenatal and transgenerational stress and AFDs have not yet been systematically studied, the present data suggest that aging processes may unmask the effects of early exposure to adverse experiences. It is important to note that even statistically small effects may have clinically important consequences and interactions [9]. The present evidence suggests that AFD effects are universal, and do not just affect developmentally compromised individuals. Interactions of various adverse experiences may provide new insights into predisposing and precipitating factors of mental illness and new or refined interventions.
